# First person – Shohei Yamamoto

**DOI:** 10.1242/bio.058621

**Published:** 2021-03-03

## Abstract

First Person is a series of interviews with the first authors of a selection of papers published in Biology Open, helping early-career researchers promote themselves alongside their papers. Shohei Yamamoto is first author on ‘Biophysical and biochemical properties of Deup1 self-assemblies: a potential driver for deuterosome formation during multiciliogenesis’, published in BiO. Shohei conducted the research described in this article while a PhD student and then postdoc in Daiju Kitagawa's lab at Department of Physiological Chemistry, Graduate School of Pharmaceutical Sciences, The University of Tokyo, Hongo, Tokyo, Japan. He is now a postdoc in the lab of Manuel Théry and Laurent Blanchoin at the Interdisciplinary Research Institute of Grenoble, CEA, Grenoble, France, investigating *in vitro* reconstitution of cytoskeleton systems.


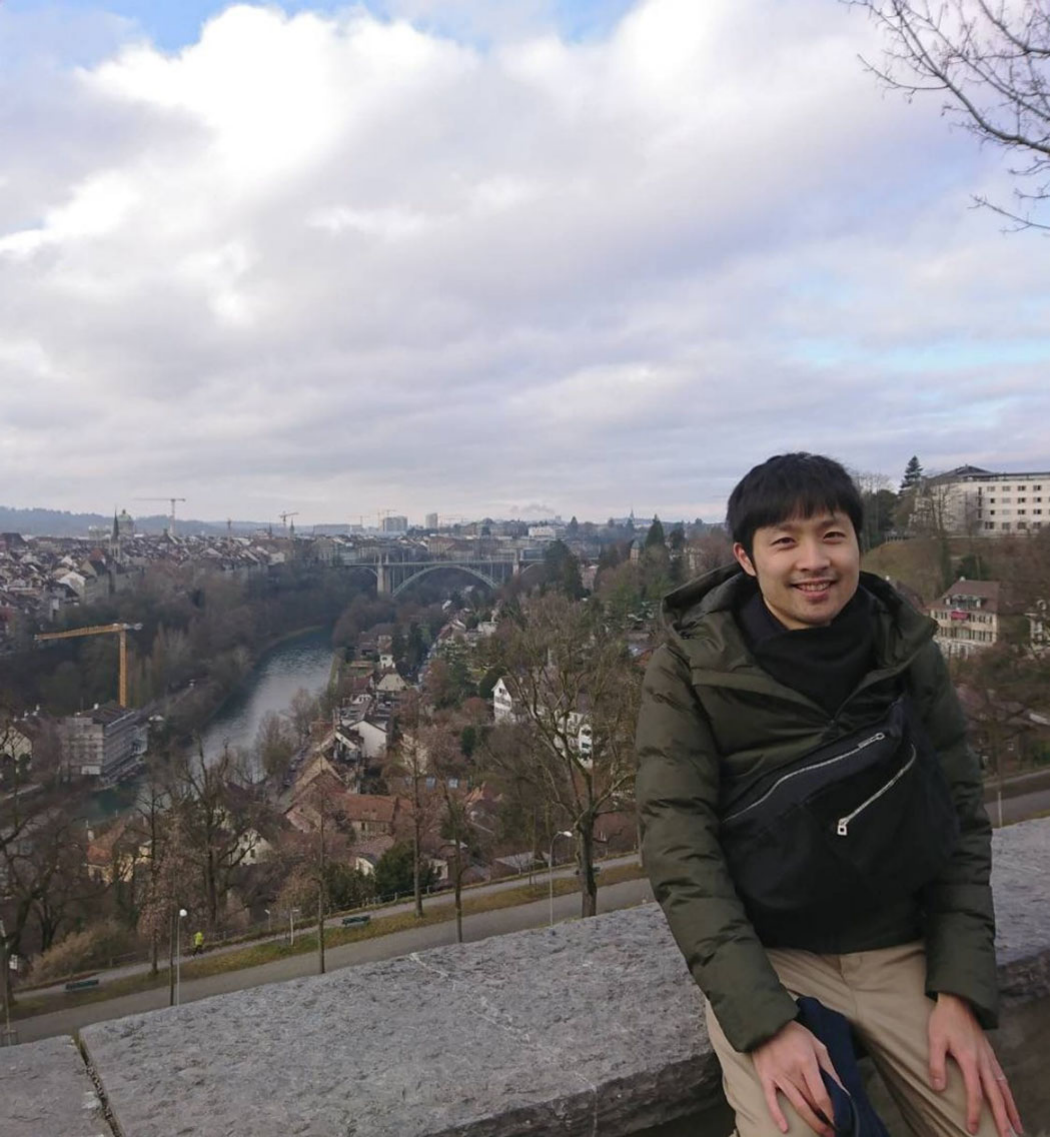


**Shohei Yamamoto**

**What is your scientific background and the general focus of your lab?**

I first studied developmental biology in Dr Yasuhiro Tomooka's lab when I was an undergraduate student. After that, I moved to the field of cell biology as a doctoral student and studied centriole biogenesis in Dr Daiju Kitagawa's lab. The lab is mainly interested in the molecular basis of centrosome organization as well as mitotic spindle formation. I prefer to combine cell biological techniques with *in vitro* reconstitution using purified proteins in order to simply understand cellular systems.

**How would you explain the main findings of your paper to non-scientific family and friends?**

Cilia are hair-like cell structures. There are several types of cells called ‘multiciliated cells’ that form multiple cilia on the cell surface. Multiciliated cells are found in our body. For example, they are present in the oviduct and support fertilization by generating extracellular fluid flow. It is not well understood how cells form multiple cilia. In the present study, we investigated the properties of an organelle called deuterosome, which is thought to support multicilia formation. We have shown that a key component of the deuterosome called Deup1 can be self-assembled into stable macromolecular structures, and that the self-assembly of Deup1 is the key to the formation of the deuterosomes. Thus, our study proposed a fundamental mechanism underlying multicilia formation.

“…our study proposed a fundamental mechanism underlying multicilia formation.”

**What has surprised you the most while conducting your research?**

During this project, I was really surprised to see the beauty of multicilia formation and centriole amplification of differentiated E1 cells. It was amazingly beautiful. When I first saw it, I couldn't take my eyes off it.

“…I was really surprised to see the beauty of multicilia formation…”

**What, in your opinion, are some of the greatest achievements in your field and how has this influenced your research?**

Dr Yasuhiro Tomooka's group established valuable cell lines including E1 cells that we used in this study. The E1 cell line is a unique cell line that has the potential to differentiate into multiciliated cells. The cell lines can be applied in many research fields. For me, this was a great opportunity to apply this cell line to my research.

**Figure:**
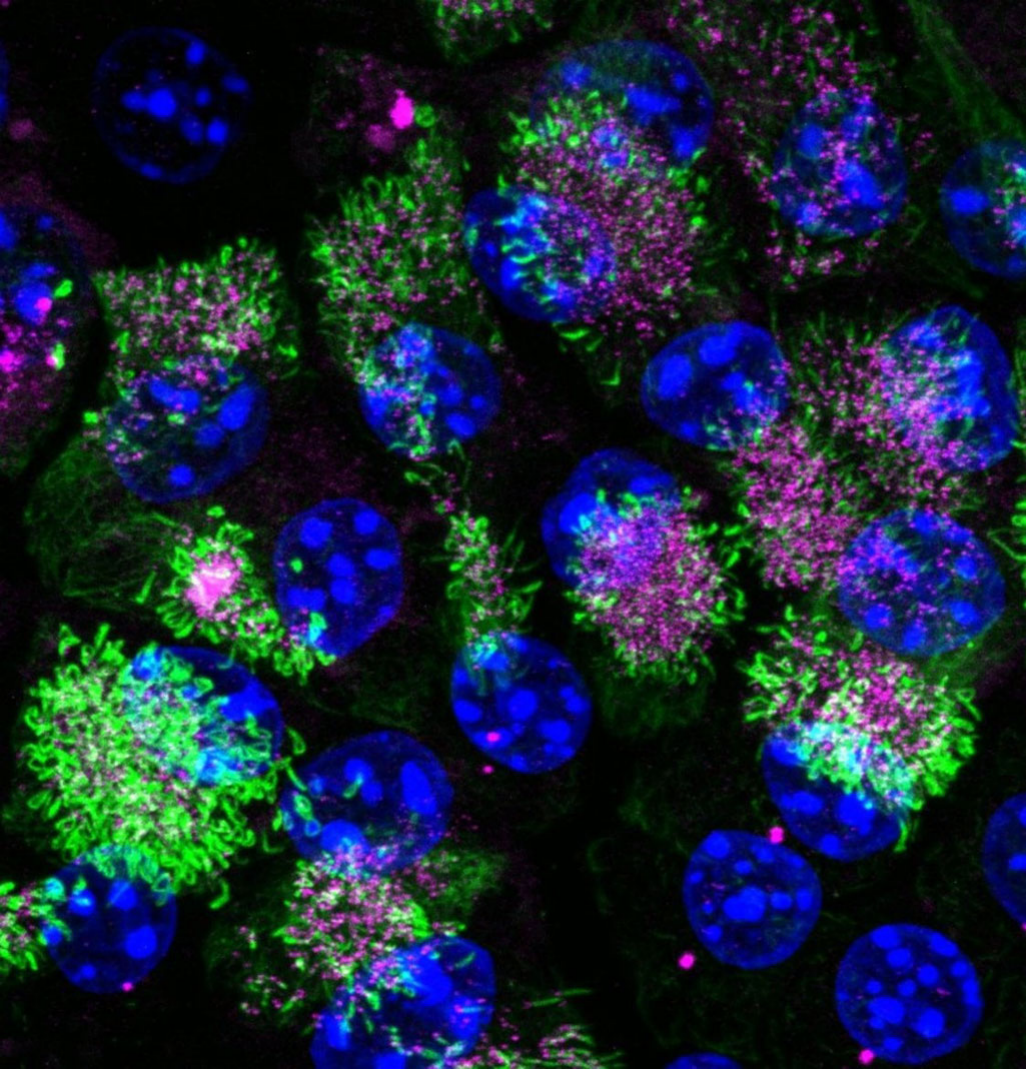
**Multiciliated cells.** Acetylated tubulin (green), γ-tubulin (magenta) and DNA (blue) were stained. Acetylated tubulin and γ-tubulin are markers of cilia and centrioles, respectively.

**What changes do you think could improve the professional lives of early-career scientists?**

To make a major breakthrough in science, we need to tackle a highly challenging project. It may require years of hard work. However, the employment of most early-career scientists is a short-term contract. Such a limitation of employment prevents us from working on highly risky projects. Therefore, improving the employment of young scientists is important to progress science.

**What's next for you?**

During my PhD, I realized that I am very interested in synthetic cell biology and then moved to the new field as a postdoc. My dream is to create artificial cells that can divide and move in order to simply understand cellular systems and to apply the technology to medicine. For such a dream, I am currently working on *in vitro* reconstitution of cytoskeletal systems and am trying to make an artificial cell model.

## References

[BIO058621C1] Yamamoto, S., Yabuki, R. and Kitagawa, D. (2021). Biophysical and biochemical properties of Deup1 self-assemblies: a potential driver for deuterosome formation during multiciliogenesis. *Biology Open.* 10, bio056432. 10.1242/bio.056432 33658185 PMC7938805

